# Steroid hormone secretion after stimulation of mineralocorticoid and NMDA receptors and cardiovascular risk in patients with depression

**DOI:** 10.1038/s41398-020-0789-7

**Published:** 2020-04-20

**Authors:** Jan Nowacki, Katja Wingenfeld, Michael Kaczmarczyk, Woo Ri Chae, Paula Salchow, Ikram Abu-Tir, Dominique Piber, Julian Hellmann-Regen, Christian Otte

**Affiliations:** Charité – Universitätsmedizin Berlin, corporate member of Freie Universität Berlin, Humboldt-Universität zu Berlin, and Berlin Institute of Health, Hindenburgdamm 30, 12203 Berlin, Germany

**Keywords:** Depression, Pathogenesis

## Abstract

Major depressive disorder (MDD) is associated with altered mineralocorticoid receptor (MR) and glucocorticoid receptor function, and disturbed glutamatergic signaling. Both systems are closely intertwined and likely contribute not only to the pathophysiology of MDD, but also to the increased cardiovascular risk in MDD patients. Less is known about other steroid hormones, such as aldosterone and DHEA-S, and how they affect the glutamatergic system and cardiovascular disease risk in MDD. We examined salivary cortisol, aldosterone, and DHEA-S secretion after stimulation of MR and glutamatergic NMDA receptors in 116 unmedicated depressed patients, and 116 age- and sex-matched healthy controls. Patients (mean age = 34.7 years, SD = ±13.3; 78% women) and controls were randomized to four conditions: (a) control condition (placebo), (b) MR stimulation (0.4 mg fludrocortisone), (c) NMDA stimulation (250 mg D-cycloserine (DCS)), and (d) combined MR/NMDA stimulation (fludrocortisone + DCS). We additionally determined the cardiovascular risk profile in both groups. DCS had no effect on steroid hormone secretion, while cortisol secretion decreased in both fludrocortisone conditions across groups. Independent of condition, MDD patients showed (1) increased cortisol, increased aldosterone, and decreased DHEA-S concentrations, and (2) increased glucose levels and decreased high-density lipoprotein cholesterol levels compared with controls. Depressed patients show profound alterations in several steroid hormone systems that are associated both with MDD pathophysiology and increased cardiovascular risk. Prospective studies should examine whether modulating steroid hormone levels might reduce psychopathology and cardiovascular risk in depressed patients.

## Introduction

Stress is a risk factor for the development of major depressive disorder (MDD)^1^ and cardiovascular disease (CVD)^2^. Furthermore, stress activates the hypothalamus–pituitary–adrenal (HPA) axis leading to the release of the steroid hormone cortisol and consecutive enhanced secretion of the neurotransmitter glutamate^3^. Both systems are closely intertwined^3,4^, and altered secretion of cortisol and glutamate is not only involved in the pathogenesis of MDD^5,6^, but may also contribute to the increased cardiovascular risk of depressed patients^1,7–9^. However, to our knowledge, steroid hormone secretion after separate or combined stimulation of the HPA axis, and glutamatergic system in depressed patients and healthy controls has not been studied so far.

Cortisol acts upon glucocorticoid receptors (GR) and mineralocorticoid receptors (MR) in the central nervous system. While GR are widely distributed in the brain, MR are predominantly expressed in the hippocampus and prefrontal cortex. MR are predominantly occupied during basal cortisol secretion, whereas GR are increasingly occupied as cortisol levels rise, for example, after stress. Cortisol binding to GR and MR inhibits HPA axis activity^1,10,11^. In MDD, this negative feedback is impaired and cortisol levels increase^12^, possibly because of impaired MR function^13–15^.

Elevated cortisol alters glutamate signaling in the hippocampus and prefrontal cortex^3^. Not surprisingly, MDD is associated with disturbed glutamatergic signaling. For example, decreased levels of glutamatergic metabolites have been reported in the medial frontal cortex of patients with MDD^6,16^. Glutamate acts on metabotropic and ionotropic receptors^3^, including the N-methyl-D-aspartate (NMDA) receptor, which has been closely implicated in the pathogenesis of MDD^17^. In fact, the U.S. Food and Drug Administration^18^ recently approved the rapid-acting NMDA receptor antagonist ketamine as a treatment for treatment-resistant depression after its efficacy was shown in several trials^19^. Importantly, ketamine strongly elevates cortisol levels^20,21^. However, the glutamate system is extremely complex and there is evidence that D-cycloserine (DCS), a partial agonist at the glycine binding site of the NMDA receptor, exhibits antidepressant effects^22,23^, and increases glutamate and GABA in the brain to the same extent as ketamine^24^.

As well as contributing to the pathogenesis of MDD, alterations in HPA activity and glutamate signaling may also contribute to the elevated risk of CVD in depressed patients^7,25,26^. Other steroid hormones, such as increased aldosterone^27,28^ and decreased DHEA-S levels^29^, are also closely linked to CVD. Importantly, increased aldosterone levels^30–32^ and decreased DHEA-S concentrations^33^ have been found in depressed patients, and both hormones interact with the glutamate system^34–36^.

Taken together, the HPA axis and the glutamatergic system play an important role in the pathogenesis of depression and might represent an important link to CVD. However, little is known about the interplay of both systems in MDD. To address this, we examined (a) salivary cortisol, aldosterone, and DHEA-S secretion after stimulation of MR and glutamatergic NMDA receptors, and (b) the cardiovascular risk profile in 116 unmedicated depressed patients and 116 age- and sex-matched healthy controls.

## Materials and methods

### Participants

In total, we examined 116 MDD patients and 116 healthy controls. We recruited patients from our in- and outpatient clinics for affective disorders (Department of Psychiatry and Psychotherapy of the Charité – Universitätsmedizin Berlin), via our website, and through flyers distributed in outpatient psychiatric practices and psychotherapy institutes. Healthy participants were recruited via our website and through flyers distributed in universities and other public spaces.

We matched depressed patients with healthy controls based on sex, age, and education duration. For every enrolled depressed patient, we recruited a control subject who was matched on these characteristics. Inclusion criteria were 18–65 years of age, a diagnosis of MDD according to the fifth edition of the Diagnostic and Statistical Manual of Mental Disorders (DSM-5)^37^, and a score of 18 or more on the Hamilton rating scale for depression (HAMD)^38^.

Exclusion criteria were intake of psychotropic medication during the last 5 days (except antidepressants as sleep medication and benzodiazepines as needed), substance abuse or dependency within the last 6 months, any current episode or history of schizophrenia, schizoaffective, or bipolar disorder (for healthy individuals, the presence of any psychiatric disorders), neuroendocrine disorders, current or past organic brain disease, acute suicidality, endocrine disorders or intake of medication with neuroendocrine effects, pregnancy or lactation, unstable cardiovascular conditions, known intolerance of study medication, or significantly abnormal laboratory values.

All participants provided written informed consent and received an expense allowance. The study was conducted in accordance with the latest version of the Declaration of Helsinki and was approved by the local ethics committee (Landesamt für Gesundheit und Soziales Berlin, 16-0031-EK 11).

### Experimental design

We used a randomized double-blind placebo-controlled parallel group design. The pharmacy of the Charité – Universitätsmedizin Berlin conducted the block randomization and blinded the medication. To stimulate MR, we used 0.4 mg fludrocortisone. To stimulate NMDA receptors, we used 250 mg DCS. In the control condition, we administered placebo. Participants were randomly assigned to one of the following four conditions: (a) control condition (placebo + placebo), (b) MR stimulation only (fludrocortisone + placebo), (c) NMDA stimulation only (placebo + DCS), and (d) combined MR and NMDA stimulation (fludrocortisone + DCS). Twenty-nine depressed patients and 29 healthy controls took part in each condition.

### Procedure

Participants were assessed for eligibility by telephone interview, and eligible participants were invited for the formal screening visit. An experienced clinician (physician or psychologist) from our team interviewed participants to acquire demographic information and to diagnose or exclude MDD according to DSM-5 criteria^37^. The HAMD interview^38^ was also conducted and participants were asked to complete the Beck Depression Inventory^39^ before undergoing an electrocardiogram. To assess the cardiovascular risk profile, we measured blood pressure and heart rate, and took blood samples for laboratory analyses. The experiment (separate or combined stimulation of MR and NMDA receptors) took place at least 24 h and not >7 days after the screening visit.

All experiments started at the same time (11:30 h) to control for influences of the circadian rhythm on cortisol secretion^40^. After arriving at the laboratory, participants rested for 30 min before the first blood pressure and heart rate measurements were taken. Two baseline saliva samples were taken at 11:55 h and 12:00 h. From 12:00 h on, we measured blood pressure and heart rate and took saliva samples every hour until 18:00 h. Participants received the first medication at 12:05 h and the second medication at 13:05 h (Supplementary Fig. 1).

At three time points (prior to medication 11:50 h, during the experiment 13:50 h, and at the end of the experiment 17:50 h), we assessed the current mood state of all participants with a visual analogue mood scale (VAMS). We asked all participants to answer the question “how are you currently feeling?” by making a cross on the VAMS, which ranged from 0 (very bad) to 100 (very good).

Between measurements, participants were allowed to walk around, read, or watch a movie. Participants were allowed to drink water, but did not eat during the experiment (11:30 h until 18:00 h). Ten minutes before every measurement, participants were asked to rest, sit on a chair, and stop drinking water. An experimenter was present during the whole testing period. Participants conducted cognitive tasks on a computer between 16:00 h and 17:00 h for ~45 min.

### Cardiovascular risk assessment

The cardiovascular risk assessment took place at the screening visit. We measured systolic and diastolic blood pressure (mmHg), and heart rate (bpm) using the Boso Medicus Uno (Bosch + Sohn, Germany) apparatus as a hemodynamometer. Blood samples were analyzed by the Labor Berlin (Charité – Universitätsmedizin Berlin). We measured total cholesterol, high-density lipoprotein (HDL) cholesterol, low-density lipoprotein (LDL) cholesterol, C-reactive protein (CRP), and glucose (all measured in mg/l or mg/dl respectively).

### Steroid hormone measurement

We collected saliva samples with Code Blue Salivettes® (Sarstedt, Germany) on the day of the experiment. Steroid levels were analyzed in the neurobiological laboratory at the Department of Psychiatry and Psychotherapy of the Charité – Universitätsmedizin Berlin. For all salivary analyses, we used enzyme-linked immunosorbent assays (ELISA; IBL International GmbH, Germany). For cortisol analyses (measured in nmol/L), an ELISA kit with a detection limit of 0.08 nmol/L was used. For aldosterone analyses (measured in pg/mL), we used an ELISA kit with a detection limit of 12 pg/mL. DHEA-S levels (measured in ng/mL) were measured using an ELISA kit optimized for saliva with a detection limit of 0.05 ng/mL. The intra-assay coefficients of variation were <8% and the inter-assay coefficients of variation were <10% for all analyses. To improve comparability, we converted all steroid hormone measurement units into pg/mL for all figures.

### Statistical analyses

Statistical analyses were conducted with IBM SPSS Statistics (version 25). Greenhouse–Geisser corrections or Welch tests were applied if assumptions of sphericity or homogeneity of variances were violated. Post hoc analyses were conducted with Bonferroni tests or contrasts if applicable.

To analyze demographic variables, we used chi-squared tests for categorical data and independent *t*-tests for continuous data. If the assumptions of the chi-squared test were violated, we used Fisher’s exact test. For cardiovascular risk assessment (blood pressure, heart rate, cholesterol, HDL cholesterol, LDL cholesterol, CRP, and glucose), we used independent *t*-tests for group comparisons.

Steroid hormone concentrations (cortisol, aldosterone, and DHEA-S) were analyzed with mixed ANOVAs with within-subject factor time (measurement time points), between-subject factors group (depressed patients and healthy controls), and condition (placebo, fludrocortisone, DCS, and fludrocortisone + DCS).

All non-normally distributed data were log transformed. Missing values for single cortisol and DHEA-S measurement time points in four participants were replaced by mean imputation (mean value of the preceding and subsequent measurement time points) to avoid loss of data.

The current mood state was analyzed with mixed ANOVAs with within-subject factor time (measurement time points), between-subject factors group (depressed patients and healthy controls), and condition (placebo, fludrocortisone, DCS, and fludrocortisone + DCS).

We calculated correlations between the cardiovascular risk assessment (blood pressure, heart rate, cholesterol, HDL cholesterol, LDL cholesterol, CRP, and glucose) measured at the screening visit and steroid hormone secretion during the experiment. For the steroid hormones cortisol, aldosterone, and DHEA-S, we calculated area under the curve values with respect to the ground.

Sample size was calculated with G*Power^41^. Effect sizes for condition effects were based on the fludrocortisone effects on cortisol (*η*^*2*^ = 0.12) reported in our earlier study^42^. The effect size (*η*^*2*^ = 0.10) for group (depressed patients versus controls) were based on a meta-analysis on differences in the cortisol response to stress between depressed patients and controls^43^. Using mixed ANOVAs with *η*^*2*^ = 0.10, *α* = 0.05, and 1 − *β* = 0.95, we calculated a total sample size of *n* = 120. To be able to find smaller effects and considering possible dropouts, we conservatively recruited a larger sample of *n* = 232 participants: *n* = 116 per group, and *n* = 58 per condition.

## Results

### Sample characteristics

Depressed patients and healthy controls did not differ in age, sex, education duration, or intake of hormonal contraceptives. There were more smokers among depressed patients than among healthy controls (Table 1). Therefore, we repeated the analyses in nonsmokers to examine a possible confounding effect of smoking status. Additional analyses on sample and depression characteristics with respect to condition are presented in the supplement (Supplementary Tables 1 and 2).

**Table 1:** Sample characteristics.

	Healthy controls	Depressed patients	Statistics
*n*	116	116	
Age, mean (*SD*)	34.9 (13.2)	34.7 (13.3)	*t*(230) = 0.1, *p* = 0.90
Women, *n* (%)	91 (78%)	91 (78%)	
Education years	12.1 (1.3)	11.8 (1.3)	*t*(230) = 1.6, *p* = 0.12
BMI	23.5 (3.4)	24.0 (4.3)	*t*(230) = −0.9, *p* = 0.36
Smoker	14 (12%)	30 (26%)	*χ²*(1) = 7.2, *p* < 0.01
Hormonal contraception	19 (21%)	19 (21%)	
HAMD	1.6 (1.3)	21.5 (3.4)	*t*(149) = −58.7, *p* < 0.001
BDI	1.4 (1.8)	25.7 (8.3)	*t*(125) = −30.8, *p* < 0.001

Values represent mean (SD) or *n* (%).

*BMI* body mass index, *HAMD* Hamilton ratings scale for depression, *BDI* Beck Depression Inventory.

Depressed patients took the following medication: benzodiazepines as needed (*n* = 13), low-dose antidepressants as sleep medication (*n* = 5), cetirizine (*n* = 1), pantoprazole (*n* = 1), ramipril (*n* = 3), lercanidipine (*n* = 1), simvastatin (*n* = 2), rosuvastatin (*n* = 1), L-thyroxine (*n* = 11), propylthiouracil (*n* = 1), dorzolamide (*n* = 1), actaea racemosa (*n* = 1), sumatriptan (*n* = 1), amlodipine (*n* = 2), indapamide (*n* = 1), valsartan (*n* = 1), and zopiclone (*n* = 2). Healthy controls took the following medication: salbutamole (*n* = 1), L-thyroxine (*n* = 10), tapentadol (*n* = 1), mesalazine (*n* = 1), ramipril (*n* = 1), metoprolol (*n* = 1), and estradiol (*n* = 1).

### Steroid hormone response to separate or combined MR and NMDA receptor stimulation

#### Cortisol

**Fig. 1:**
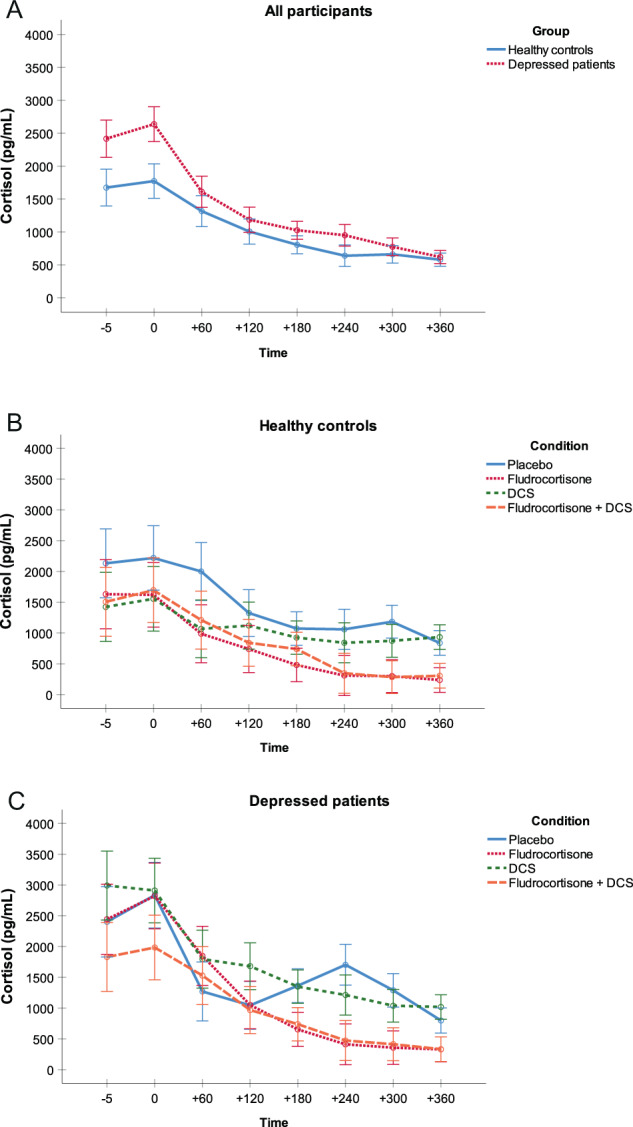
Cortisol secretion. Mean cortisol secretion in pg/mL (SE) for **a** both groups across conditions, **b** healthy controls for each condition, and **c** depressed patients for each condition.

We found a main effect of group on cortisol levels (*F*(1,222) = 4.0, *p* < 0.05, *η*^*2*^ = 0.02), indicating that depressed patients had higher cortisol concentrations compared with healthy controls independent of condition and time (Fig. 1a). In addition, we found a main effect of condition (*F*(3,222) = 4.8, *p* < 0.01, *η*^*2*^ = 0.06) and time (*F*(3,696) = 266.2, *p* < 0.001, *η*^*2*^ = 0.55) on cortisol concentrations, and an interaction between condition × time (*F*(9,696) = 11.0, *p* < 0.001, *η*^*2*^ = 0.13). One-way ANOVAs with Bonferroni post hoc tests revealed decreased cortisol secretion in both fludrocortisone conditions compared with the placebo and DCS-only conditions (all *p* < 0.05; Fig. 1b, c). Analyses in nonsmokers confirmed the results.

#### Aldosterone

**Fig. 2:**
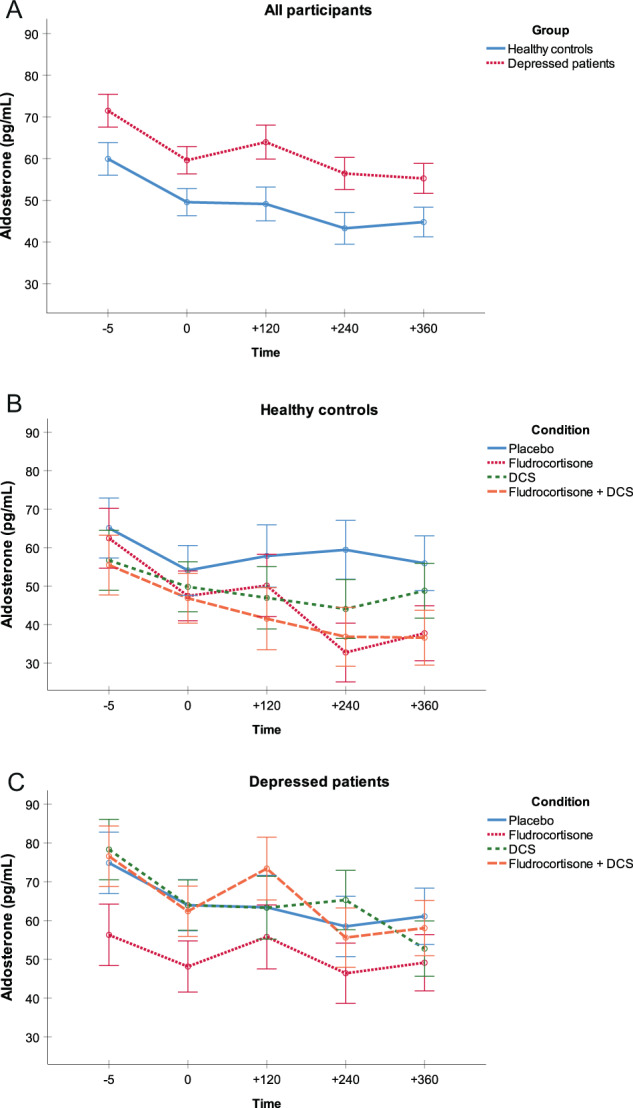
Aldosterone secretion. Mean aldosterone secretion in pg/mL (SE) for **a** both groups across conditions, **b** healthy controls for each condition, and **c** depressed patients for each condition.

We found a main effect of group on aldosterone levels (*F*(1,222) = 10.2, *p* < 0.01, *η*^*2*^ = 0.04), indicating that depressed patients had higher aldosterone concentrations compared with healthy controls independent of condition and time (Fig. 2a). We found no main effect of condition (*p* > 0.05; Fig. 2b, c) but a main effect of time (*F*(2,660) = 33.7, *p* < 0.001, *η*^*2*^ = 0.13) on aldosterone levels, indicating a decrease in aldosterone concentrations. Analyses in nonsmokers confirmed the results.

#### DHEA-S

**Fig. 3:**
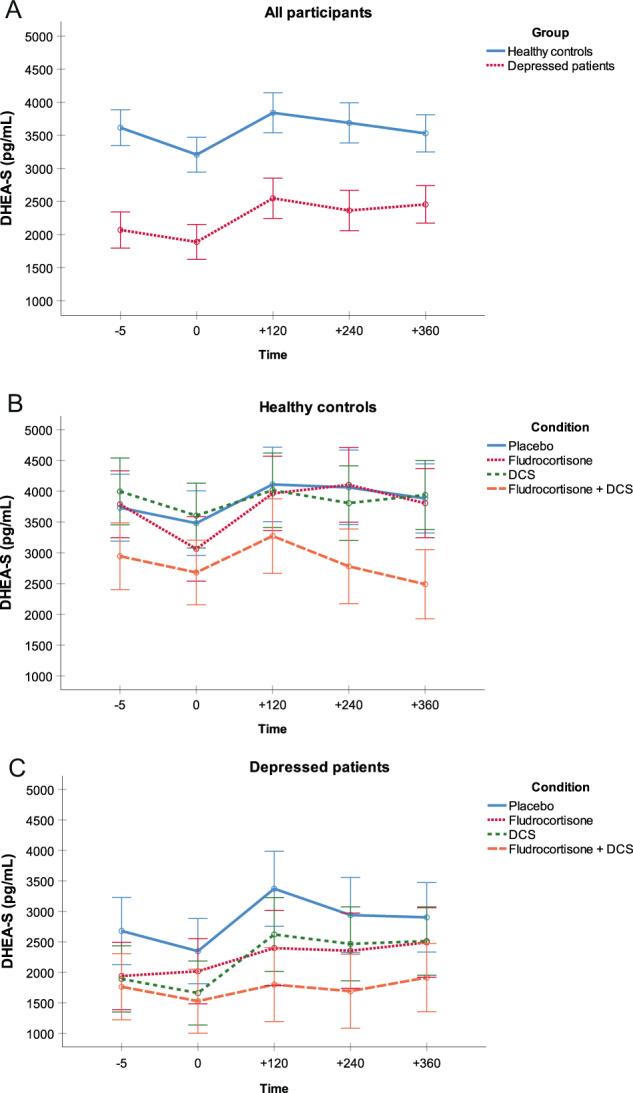
DHEA-S secretion. Mean DHEA-S secretion in pg/mL (SE) for **a** both groups across conditions, **b** healthy controls for each condition, and **c** depressed patients for each condition.

There was a main effect of group on DHEA-S concentrations (*F*(1,222) = 8.3, *p* < 0.01, *η*^*2*^ = 0.04), indicating that depressed patients had lower DHEA-S concentrations compared with healthy controls independent of condition and time (Fig. 3a). We found no main effect of condition (*p* > 0.05; Fig. 3b, c) but a main effect of time (*F*(3,704) = 12.3, *p* < 0.001, *η*^*2*^ = 0.05) on DHEA-S concentrations. Analyses in nonsmokers revealed a slightly reduced effect size for the main effect of group (*F*(1,179) = 3.7, *p* = 0.056, *η*^*2*^ = 0.02) and confirmed the time effect (*p* < 0.001).

#### Blood pressure and heart rate

For systolic blood pressure, we found a main effect of time (*F*(5,1150) = 43.6, *p* < 0.001, *η*^*2*^ = 0.16) and a condition × time interaction (*F*(15,1150) = 2.4, *p* < 0.01, *η*^*2*^ = 0.31), indicating an increase in systolic blood pressure within each condition over time (Supplementary Fig. 2). There was a main effect of time on diastolic blood pressure (*F*(5,1205) = 44.6, *p* < 0.001, *η*^*2*^ = 0.17) and a condition × time interaction (*F*(16,1205) = 2.9, *p* < 0.001, *η*^*2*^ = 0.37), indicating an overall increase in diastolic blood pressure within each condition over time (Supplementary Fig. 3).

We found a main effect of group on heart rate (*F*(1,224) = 15.4, *p* < 0.001, *η*^*2*^ = 0.06) and a main effect of time on heart rate (*F*(4,1025) = 188.5, *p* < 0.001, *η*^*2*^ = 0.46). In addition, we found a group × condition interaction (*F*(3,224) = 2.9, *p* < 0.05, *η*^*2*^ = 0.04), indicating increased heart rate in depressed patients compared with healthy controls within the fludrocortisone-only condition (Supplementary Fig. 4). Analyses in nonsmokers confirmed these results.

#### Mood assessment

We found a main effect of group on current mood state (*F*(1,222) = 200.0, *p* < 0.01, *η*^*2*^ = 0.47), indicating that depressed patients were in a worse mood compared with healthy controls (Supplementary Fig. 5). However, there was neither a main effect of condition or time nor a condition × group interaction. We found an interaction between group × time (*F*(1,378) = 10.5, *p* < 0.001, *η*^*2*^ = 0.05), indicating that subjective mood ratings of depressed patients slightly increased during the experiment, while healthy individuals exhibited a slight decrease in subjective mood (Supplementary Fig. 5).

#### Cardiovascular risk

Depressed patients and healthy controls did not differ in blood pressure, heart rate, total cholesterol, LDL cholesterol, and CRP. However, we found lower HDL cholesterol and higher glucose levels in depressed patients than in healthy controls (Table 2). Analyses in the group of nonsmokers confirmed the results and also showed higher diastolic blood pressure in depressed patients (*M* = 80.6, SD = 7.3) than in healthy controls (*M* = 77.9, SD = 8.7; *p* < 0.05).

**Table 2:** Cardiovascular risk in depressed patients and healthy controls.

	Healthy controls	Depressed patients	Statistics
Systolic blood pressure	119.4 (13.2)	119.1 (12.1)	*t*(230) = 0.2, *p* = 0.87
Diastolic blood pressure	78.4 (8.5)	79.7 (7.2)	*t*(223) = −1.2, *p* = 0.22
Heart rate	71.6 (10.8)	73.7 (10.0)	*t*(230) = −1.5, *p* = 0.12
Total cholesterol	181.2 (35.6)	184.6 (39.1)	*t*(229) = −0.7, *p* = 0.49
HDL cholesterol	69.4 (19.6)	64.1 (17.4)	*t*(229) = 2.1, *p* = 0.03*
LDL cholesterol	108.0 (33.2)	112.4 (33.3)	*t*(229) = −1.0, *p* = 0.32
CRP	1.6 (3.6)	1.8 (2.8)	*t*(229) = −0.5, *p* = 0.59
Glucose	84.2 (14.8)	88.3 (13.1)	*t*(230) = −2.2, *p* = 0.03*

Values represent mean (SD) and significant differences are marked (*).

*HDL cholesterol* high-density lipoprotein cholesterol, *LDL cholesterol* low-density lipoprotein cholesterol, *CRP* C-reactive protein.

### Correlational analyses

We calculated correlations between cardiovascular risk variables and steroid hormone values. To control for multiple testing, we performed Bonferroni corrections (*p* = 0.0018, after 0.05/27). We found a correlation between cortisol and aldosterone (*r* = 0.34, *p* < 0.001), but no other correlations were significant.

## Discussion

The aim of this study was to examine salivary cortisol, aldosterone, and DHEA-S secretion in depressed patients and healthy controls after stimulation of MR with fludrocortisone and glutamatergic NMDA receptors with DCS. We also examined CVD risk in these participants. We report four main results: (1) NMDA receptor stimulation with DCS did not affect steroid hormone secretion in depressed patients and healthy individuals, (2) MR stimulation with fludrocortisone inhibited cortisol secretion across groups, (3) depressed patients showed higher cortisol and aldosterone values, but lower DHEA-S concentrations compared with healthy individuals independent of condition, and (4) depressed patients had higher glucose levels and lower HDL cholesterol values than healthy controls.

There is evidence for a bidirectional association between glutamate signaling and the HPA axis. For example, stress and elevated cortisol levels increase glutamate signaling in the hippocampus and prefrontal cortex^3^. Conversely, the NMDA receptor antagonist ketamine activates the HPA axis and elevates cortisol levels^20,21,44,45^. However, in this study, the partial NMDA receptor agonist DCS did not affect cortisol secretion, which is in line with earlier studies^46–48^. Interestingly, a previous study showed that ketamine but not memantine (another NMDA receptor antagonist) increases cortisol secretion^44^. These findings suggest that ketamine increases cortisol secretion independently of the NMDA receptor. DCS has attracted tremendous interest in neuroscience research because of its role in learning, neuroplasticity, memory, and as a potential antidepressant^22,49,50^. Our results suggest that these effects are independent of HPA activity.

NMDA receptor stimulation did not affect hormone secretion, but the MR agonist fludrocortisone inhibited cortisol (but not aldosterone or DHEA-S) across groups. It is well known that MR stimulation inhibits HPA activity^51,52^. In accordance with our previous findings, the cortisol responses to fludrocortisone did not differ between young, unmedicated depressed patients and healthy individuals in the current study^53^. This suggests that MR function is intact in these patients. However, Lembke et al.^14^ found attenuated MR-mediated inhibition of cortisol secretion in patients with psychotic depression. Furthermore, Juruena et al.^13^ observed diminished MR function in patients with treatment-resistant depression. Given the well-established glucocorticoid resistance in depressed patients^54^, the authors speculated that patients with treatment-resistant depression (and potentially patients with psychotic depression) are not able to compensate for GR resistance by increasing MR function. However, our findings suggest that this might be possible in less severely depressed patients.

The third main result was that depressed patients had higher cortisol and aldosterone values, but lower DHEA-S concentrations than healthy individuals. To our knowledge, this is the first study to demonstrate alterations of three important steroid hormones in the same patients. Importantly, all of these hormones are stress responsive^27,55^ and have been associated with depression^5,30,33,56,57^. While increased cortisol secretion in depressed patients is well established^5,12^, only few studies have examined aldosterone secretion in MDD. However, these studies have consistently found increased aldosterone levels in depressed patients compared with healthy individuals^30,57^. Furthermore, salivary aldosterone was associated with depression severity^58^ and predicted treatment response to standard antidepressants^59,60^. In turn, patients with primary aldosteronism exhibit more depressive symptoms compared with the general population^61,62^. Finally, animal studies demonstrated that aldosterone increases earlier than corticosterone after induction of depressive behavior^63^ and that subchronic treatment with aldosterone induces depression-like behaviors in rats^64^. In sum, these studies suggest that aldosterone is closely involved in the pathophysiology of MDD.

With our cross-sectional design, we cannot determine causality between depression and altered steroid hormone secretion. However, there is strong evidence that alterations in steroid hormones contribute to the development of depression. For example, childhood adversity is associated with altered cortisol and DHEA secretion^55^, which in turn increases the risk of depression^65,66^. Furthermore, aldosterone induces depressive symptoms in animals^64,67^. On the other hand, depression itself can alter steroid hormone concentrations. For example, the lifestyle of depressed patients (such as poor diet, reduced sleep, and less physical activity) affects steroid hormone secretion^68^. Therefore, the association between depression and altered steroid secretion is likely bidirectional, leading to a vicious circle of more severe depression and more profound disturbances in steroid hormone signaling.

These alterations in steroid hormone secretion likely contribute to the increased cardiovascular risk in depressed patients. Indeed, we show in the present study that depressed patients had higher glucose levels and lower HDL cholesterol values than healthy controls. Increased aldosterone is an established risk factor for mortality in CVD, and blocking MR has beneficial effects on many CVD endpoints, including mortality^69,70^. Several prospective studies in different populations have shown that higher cortisol values are associated with cardiovascular mortality^71–73^. In addition, a meta-analysis of 25 studies showed an association between low DHEA-S levels and increased mortality in patients with CVD^29^. Therefore, our findings have strong clinical implications because they suggest that these endocrine alterations in depressed patients contribute to their increased CVD risk and their increased mortality. In our cross-sectional study, however, there was no correlation between any steroid hormone and any CVD risk factor. One explanation might be that we examined a relatively young population of unmedicated depressed patients and healthy controls, who did not suffer (yet) from severe metabolic and/or cardiovascular conditions. Indeed, our participants were younger and less physically impaired compared with participants of studies that found an association between steroid hormones and CVD^74^, or an association between steroid hormones and cardiovascular mortality^71–73^. Future longitudinal studies should examine whether modulating these endocrine systems can improve CVD risk and psychopathology in depressed patients. Randomized controlled trials have revealed encouraging evidence that DHEA both decreases CVD risk and improves depressive symptoms^75,76^.

Our study had several limitations. First, in our sample there were much more women than men (78% women). Thus, our results cannot be generalized to men. Furthermore, we studied a comparably young population of depressed patients with few medical comorbidities, so our results cannot be generalized to older people and patient groups with severe medical conditions. However, the homogeneous nature of our group of depressed patients is also a strength as it increases internal validity. Second, there was no specific time of day when we collected the plasma samples and, therefore, our blood samples were not restricted to fasting glucose or fasting lipids, but included non-fasting values as well. However, according to the consensus statement from the European Atherosclerosis Society and European Federation of Clinical Chemistry and Laboratory Medicine there is no clinically relevant difference between fasting and non-fasting lipid measurements^77^. Furthermore, there is also evidence that non-fasting glucose values are associated with incident CVD^78,79^. Overall, the clinical significance of non-fasting glucose and non-fasting lipid values seems to be established. Third, we did not measure HbA1c as a long-term marker of glucose concentrations. Fourth, due to the limited quantity of saliva, we were restricted to three steroids. Therefore, we chose those steroids for which several earlier studies had been shown an association with MDD^5,30,33,56,57^. The plasma concentrations of the sulfated form (DHEA-S) is between 250 and 500 times higher (women and men, respectively) than the concentrations of DHEA^80^. In addition, both steroid hormones are correlated^81,82^. Therefore, we believe that measuring DHEA-S also provides a reliable assessment of DHEA values. Fifth, we used 250 mg of DCS, which is considered a moderate dosage that can lead to partial agonism of the NMDA receptor. However, it has been suggested that DCS acts as an NMDA receptor antagonist at high doses in the range of 750–1000 mg (ref. ^17^). The NMDA receptor antagonist ketamine increases cortisol secretion^20,21^. However, the NMDA receptor antagonist memantine does not^44^. We cannot exclude that a higher dosage of DCS that acts as an NMDA receptor antagonist would have affected steroid hormone secretion and further studies should examine this question. Sixth, while the MR affinity of fludrocortisone is ~150 times higher than its GR affinity^83^, fludrocortisone has some glucocorticoid potency. The extent of its glucocorticoid potency ranges from negligible to rather moderate depending on the source of the literature and variable being examined^84,85^. Thus, remaining GR activity could have contributed to the effects of fludrocortisone in our study. Finally, even though we recruited a relatively large sample (*n* = 232), we still might have lacked power to find an association between NMDA receptor stimulation and steroid secretion even though the effect sizes were small and presumably clinically irrelevant.

Strengths of the study include the lack of antidepressive treatment during the study, careful matching of healthy individuals to depressed patients based on age, sex, and years of education, and strongly controlled experimental conditions during saliva collection with almost no missing data. In addition, the demographic characteristics did not differ between participants across the four conditions, except for fewer male participants in the fludrocortisone + DCS condition compared with other conditions. However, our main findings were independent of condition—MDD patients showed increased cortisol, increased aldosterone, and decreased DHEA-S concentrations, and increased glucose levels and decreased HDL cholesterol levels compared with controls. Therefore, the differences in sex distribution across the four conditions likely did not affect the main results.

In conclusion, we found that steroid hormone alterations and cardiovascular risk are higher in patients with depression than in healthy individuals. Future research should prospectively examine whether manipulating these steroid systems can improve the symptoms and cardiovascular risk of patients with depression.

## Supplementary information

Supplemental information: titles and legends

Table S1

Table S2

Figure S1

Figure S2

Figure S3

Figure S4

Figure S5

## References

[1] Otte C (2016). Major depressive disorder. Nat. Rev. Dis. Prim..

[2] Brotman DJ, Golden SH, Wittstein IS (2007). The cardiovascular toll of stress. Lancet.

[3] Popoli M, Yan Z, McEwen BS, Sanacora G (2012). The stressed synapse: the impact of stress and glucocorticoids on glutamate transmission. Nat. Rev. Neurosci..

[4] Treccani G (2014). Stress and corticosterone increase the readily releasable pool of glutamate vesicles in synaptic terminals of prefrontal and frontal cortex. Mol. Psychiatry.

[5] Stetler C, Miller GE (2011). Depression and hypothalamic-pituitary-adrenal activation: a quantitative summary of four decades of research. Psychosom. Med..

[6] Murrough JW, Abdallah CG, Mathew SJ (2017). Targeting glutamate signalling in depression: progress and prospects. Nat. Rev. Drug Discov..

[7] Penninx BWJH (2017). Depression and cardiovascular disease: epidemiological evidence on their linking mechanisms. Neurosci. Biobehav. Rev..

[8] Nemeroff CB, Goldschmidt-Clermont PJ (2012). Heartache and heartbreak—the link between depression and cardiovascular disease. Nat. Rev. Cardiol..

[9] Carney RM, Freedland KE (2016). Depression and coronary heart disease. Nat. Rev. Cardiol..

[10] ter Heegde F, De Rijk RH, Vinkers CH (2015). The brain mineralocorticoid receptor and stress resilience. Psychoneuroendocrinology.

[11] de Kloet, E., Meijer, O., de Nicola, A., de Rijk, R. & Joëls, M. Importance of the brain corticosteroid receptor balance in metaplasticity, cognitive performance and neuro-inflammation. *Front. Neuroendocrinol.* **49**, 124–145 (2018). 10.1016/j.yfrne.2018.02.003 29428549

[12] Pariante CM, Lightman SL (2019). The HPA axis in major depression: classical theories and new developments. Trends Neurosci..

[13] Juruena MF (2013). The role of mineralocorticoid receptor function in treatment-resistant depression. J. Psychopharmacol..

[14] Lembke A (2013). The mineralocorticoid receptor agonist, fludrocortisone, differentially inhibits pituitary–adrenal activity in humans with psychotic major depression. Psychoneuroendocrinology.

[15] Hinkelmann K (2016). Mineralocorticoid receptor function in depressed patients and healthy individuals. Prog. Neuro-Psychopharmacol. Biol. Psychiatry.

[16] Moriguchi, S. et al. Glutamatergic neurometabolite levels in major depressive disorder: a systematic review and meta-analysis of proton magnetic resonance spectroscopy studies. *Mol. Psychiatry* **24**, 952–964 (2019). 10.1038/s41380-018-0252-9 PMC6755980 30315224

[17] Chan SY, Matthews E, Burnet PW (2017). ON or OFF?: modulating the N-methyl-D-aspartate receptor in major depression. Front. Mol. Neurosci..

[18] Food and Drug Administration. *FDA Approves New Nasal Spray Medication for Treatment-resistant Depression; Available Only at a Certified Doctor’s Office or Clinic*. Retrieved June 11 (2019) from.

[19] Krystal JH, Abdallah CG, Sanacora G, Charney DS, Duman RS (2019). Ketamine: a paradigm shift for depression research and treatment. Neuron.

[20] Khalili-Mahani N, Martini CH, Olofsen E, Dahan A, Niesters M (2015). Effect of subanaesthetic ketamine on plasma and saliva cortisol secretion. Br. J. Anaesth..

[21] Khalili-Mahani N (2015). Ketamine interactions with biomarkers of stress: a randomized placebo-controlled repeated measures resting-state fMRI and PCASL pilot study in healthy men. NeuroImage.

[22] Heresco-Levy U (2013). A randomized add-on trial of high-dose D-cycloserine for treatment-resistant depression. Int. J. Neuropsychopharmacol..

[23] Schade S, Paulus W (2016). D-Cycloserine in neuropsychiatric diseases: a systematic review.. Int. J. Neuropsychopharmacol.

[24] Kantrowitz JT, Milak MS, Mao X, Shungu DC, Mann JJd-Cycloserine (2016). an NMDA glutamate receptor glycine site partial agonist, induces acute increases in brain glutamate plus glutamine and gaba comparable to ketamine. Am. J. Psychiatry.

[25] Whooley MA, Wong JM (2013). Depression and cardiovascular disorders. Annu. Rev. Clin. Psychol..

[26] Zheng Y (2016). Metabolites of glutamate metabolism are associated with incident cardiovascular events in the PREDIMED PREvencion con DIeta MEDiterranea (PREDIMED) Trial. J. Am. Heart Assoc..

[27] Kubzansky LD, Adler GK (2010). Aldosterone: A forgotten mediator of the relationship between psychological stress and heart disease. Neurosci. Biobehav. Rev..

[28] Dahal K (2018). Aldosterone antagonist therapy and mortality in patients with ST-segment elevation myocardial infarction without heart failure: a systematic review and meta-analysis. JAMA Intern. Med..

[29] Wu TT (2017). Prognostic value of dehydroepiandrosterone sulfate for patients with cardiovascular disease: a systematic review and meta‐analysis. J. Am. Heart Assoc..

[30] Emanuele E, Geroldi D, Minoretti P, Coen E, Politi P (2005). Increased plasma aldosterone in patients with clinical depression. Arch. Med. Res..

[31] Murck H, Büttner M, Kircher T, Konrad C (2014). Genetic, molecular and clinical determinants for the involvement of aldosterone and its receptors in major depression. Nephron. Physiol..

[32] Häfner S (2012). To live alone and to be depressed, an alarming combination for the renin–angiotensin–aldosterone-system (RAAS). Psychoneuroendocrinology.

[33] Hu Q (2015). Clinical significance of decreased protein expression of dehydroepiandrosterone sulfate in the development of depression: a meta-analysis. J. Affect. Disord..

[34] Gabor A, Leenen FH (2013). Central mineralocorticoid receptors and the role of angiotensin II and glutamate in the paraventricular nucleus of rats with angiotensin II–induced hypertension. Hypertension.

[35] Zoupa E, Gravanis A, Pitsikas N (2019). The novel dehydroepiandrosterone (DHEA) derivative BNN27 counteracts behavioural deficits induced by the NMDA receptor antagonist ketamine in rats. Neuropharmacology.

[36] Zaric M (2018). Regional-specific effects of cerebral ischemia/reperfusion and dehydroepiandrosterone on synaptic NMDAR/PSD-95 complex in male Wistar rats. Brain Res..

[37] Association A. P. *Diagnostic and statistical manual of mental disorders (DSM-5®)* (American Psychiatric Pub, 2013). 10.1590/s2317-17822013000200017 24413388

[38] Hamilton M. *The Hamilton rating scale for depression. Assessment of depression* 143–152 (Springer, 1986).

[39] Beck AT, Ward C, Mendelson M, Mock J, Erbaugh J (1961). Beck depression inventory (BDI). Arch. Gen. Psychiatry.

[40] Edwards S, Clow A, Evans P, Hucklebridge F (2001). Exploration of the awakening cortisol response in relation to diurnal cortisol secretory activity. Life Sci..

[41] Faul F, Erdfelder E, Lang A-G, Buchner A (2007). G* Power 3: a flexible statistical power analysis program for the social, behavioral, and biomedical sciences. Behav. Res. methods.

[42] Schultebraucks K (2016). Selective attention to emotional cues and emotion recognition in healthy subjects: the role of mineralocorticoid receptor stimulation. Psychopharmacology.

[43] Burke HM, Davis MC, Otte C, Mohr DC (2005). Depression and cortisol responses to psychological stress: a meta-analysis. Psychoneuroendocrinology.

[44] Hergovich N (2001). Comparison of the effects of ketamine and memantine on prolactin and cortisol release in men: a randomized, double-blind, placebo-controlled trial. Neuropsychopharmacology.

[45] Krystal JH (1994). Subanesthetic effects of the noncompetitive NMDA antagonist, ketamine, in humans: psychotomimetic, perceptual, cognitive, and neuroendocrine responses. Arch. Gen. Psychiatry.

[46] van Berckel BN (1998). The partial NMDA agonist D-cycloserine stimulates LH secretion in healthy volunteers. Psychopharmacology.

[47] van Berckel BN (1997). Behavioral and neuroendocrine effects of the partial NMDA agonist D-cycloserine in healthy subjects. Neuropsychopharmacology.

[48] Feld GB, Lange T, Gais S, Born J (2013). Sleep-dependent declarative memory consolidation—unaffected after blocking NMDA or AMPA receptors but enhanced by NMDA coagonist D-cycloserine. Neuropsychopharmacology.

[49] Otto MW (2016). Enhancement of psychosocial treatment with d-cycloserine: models, moderators, and future directions. Biol. Psychiatry.

[50] Peyrovian B (2019). The glycine site of NMDA receptors: a target for cognitive enhancement in psychiatric disorders. Prog. Neuro-Psychopharmacol. Biol. Psychiatry.

[51] Wingenfeld K, Otte C (2019). Mineralocorticoid receptor function and cognition in health and disease. Psychoneuroendocrinology.

[52] de Kloet ER, de Kloet SF, de Kloet CS, de Kloet AD (2019). Top‐down and bottom‐up control of stress‐coping. J. Neuroendocrinol..

[53] Otte C (2015). Mineralocorticoid receptor stimulation improves cognitive function and decreases cortisol secretion in depressed patients and healthy individuals. Neuropsychopharmacology.

[54] Pariante CM (2017). Why are depressed patients inflamed? A reflection on 20 years of research on depression, glucocorticoid resistance and inflammation. Eur. Neuropsychopharmacol..

[55] Kamin HS, Kertes DA (2017). Cortisol and DHEA in development and psychopathology. Hormones Behav..

[56] Knorr U, Vinberg M, Kessing LV, Wetterslev J (2010). Salivary cortisol in depressed patients versus control persons: a systematic review and meta-analysis. Psychoneuroendocrinology.

[57] Murck H (2003). The renin-angiotensin-aldosterone system in patients with depression compared to controls–a sleep endocrine study. BMC Psychiatry.

[58] Segeda V, Izakova L, Hlavacova N, Bednarova A, Jezova D (2017). Aldosterone concentrations in saliva reflect the duration and severity of depressive episode in a sex dependent manner. J. Psychiatr. Res..

[59] Büttner M (2015). Target-based biomarker selection–mineralocorticoid receptor-related biomarkers and treatment outcome in major depression. J. Psychiatr. Res..

[60] Murck H, Braunisch MC, Konrad C, Jezova D, Kircher T (2019). Markers of mineralocorticoid receptor function: changes over time and relationship to response in patients with major depression. Int. Clin. Psychopharmacol..

[61] Velema MS (2017). Health-related quality of life and mental health in primary aldosteronism: a systematic review. Horm. Metab. Res..

[62] Künzel HE (2012). Psychopathological symptoms in patients with primary hyperaldosteronism – possible pathways. Horm. Metab. Res..

[63] Franklin M, Bermudez I, Murck H, Singewald N, Gaburro S (2012). Sub-chronic dietary tryptophan depletion – an animal model of depression with improved face and good construct validity. J. Psychiatr. Res..

[64] Hlavacova N (2012). Subchronic treatment with aldosterone induces depression-like behaviours and gene expression changes relevant to major depressive disorder. Int. J. Neuropsychopharmacol..

[65] Harris T (2000). Morning cortisol as a risk factor for subsequent major depressive disorder in adult women. Br. J. Psychiatry.

[66] Goodyer IM, Herbert J, Tamplin A, Altham P (2000). Recent life events, cortisol, dehydroepiandrosterone and the onset of major depression in high-risk adolescents. Br. J. Psychiatry.

[67] Franklin M (2015). Aldosterone signals the onset of depressive behaviour in a female rat model of depression along with SSRI treatment resistance. Neuroendocrinology.

[68] Lopresti AL, Hood SD, Drummond PD (2013). A review of lifestyle factors that contribute to important pathways associated with major depression: diet, sleep and exercise. J. Affect. Disord..

[69] Gomez-Sanchez E (2016). Third generation mineralocorticoid receptor antagonists; why we need a fourth. J. Cardiovascular Pharmacol..

[70] DuPont JJ, Jaffe IZ (2017). 30 YEARS OF THE MINERALOCORTICOID RECEPTOR: the role of the mineralocorticoid receptor in the vasculature. J. Endocrinol..

[71] Kumari M, Shipley M, Stafford M, Kivimaki M (2011). Association of diurnal patterns in salivary cortisol with all-cause and cardiovascular mortality: findings from the Whitehall II study. J. Clin. Endocrinol. Metab..

[72] Vogelzangs N (2010). Urinary cortisol and six-year risk of all-cause and cardiovascular mortality. J. Clin. Endocrinol. Metab..

[73] Hammer F (2016). High evening salivary cortisol is an independent predictor of increased mortality risk in patients with systolic heart failure. Int. J. Cardiol..

[74] Buglioni A (2015). Circulating aldosterone and natriuretic peptides in the general community. Hypertension.

[75] Schmidt PJ (2005). Dehydroepiandrosterone monotherapy in midlife-onset major and minor depression. Arch. Gen. Psychiatry.

[76] Weiss EP, Villareal DT, Fontana L, Han D-H, Holloszy JO (2011). Dehydroepiandrosterone (DHEA) replacement decreases insulin resistance and lowers inflammatory cytokines in aging humans. Aging (Albany NY).

[77] Nordestgaard BG (2016). Fasting is not routinely required for determination of a lipid profile: clinical and laboratory implications including flagging at desirable concentration cut-points-a joint consensus statement from the European Atherosclerosis Society and European Federation of Clinical Chemistry and Laboratory Medicine. Eur. Heart J..

[78] Benn M (2012). Nonfasting glucose, ischemic heart disease, and myocardial infarction: a Mendelian randomization study. J. Am. Coll. Cardiol..

[79] Imano H (2012). Non-fasting blood glucose and risk of incident coronary heart disease in middle-aged general population: The Circulatory Risk in Communities Study (CIRCS). Preventive Med..

[80] Webb SJ, Geoghegan TE, Prough RA, Michael Miller KK (2006). The biological actions of dehydroepiandrosterone involves multiple receptors. Drug Metab. Rev..

[81] Straub RH (1998). Serum dehydroepiandrosterone (DHEA) and DHEA sulfate are negatively correlated with serum interleukin-6 (IL-6), and DHEA inhibits IL-6 secretion from mononuclear cells in man in vitro: possible link between endocrinosenescence and immunosenescence. J. Clin. Endocrinol. Metab..

[82] Folan MM (2001). Dehydroepiandrosterone, dehydroepiandrosterone-sulfate, and cortisol concentrations in intensive care unit patients. Crit. Care Med..

[83] Agarwal M, Coupry F, Philippe M (1977). Physiological activity and receptor binding of 9α fluorohydrocortisone. Biochem. Biophys. Res. Commun..

[84] Grossmann C (2004). Transactivation via the human glucocorticoid and mineralocorticoid receptor by therapeutically used steroids in CV-1 cells: a comparison of their glucocorticoid and mineralocorticoid properties. Eur. J. Endocrinol..

[85] Miller D. Adrenocorticoids. in *Foye’s Principles of Medicinal Chemistry*, 6th edn, (eds Lemke, T. L. & Williams, D. A.) 890–891 (Lippincott Williams & Wilkins, a Wolters Kluwer business: Baltimore, MD, 2008).

