# Supplementary material for: Steroid hormone secretion after stimulation of mineralocorticoid and NMDA receptors and cardiovascular risk in patients with depression
Source: Transl Psychiatry. 2020 Apr 20;10:109. doi: 10.1038/s41398-020-0789-7 (PMC7171120; doi:10.1038/s41398-020-0789-7)
Supplement: Supplementary file 1 — Supplemental information: titles and legends [file 41398_2020_789_MOESM1_ESM.docx]

# Supplemental information

# Table titles and legends

**Table S1**. Sample characteristics separated for each condition

Legend: FLU = Fludrocortisone; DCS = D-cycloserine; BMI = Body mass index; HAMD = Hamilton Ratings Scale for Depression; BDI = Beck Depression Inventory; Values represent mean (SD) or n (%).

**Table S2**. Depression characteristics for the whole sample and four conditions

Legend: FLU = Fludrocortisone; DCS = D-cycloserine; in the fludrocortisone condition, specifier information was missing for two participants. FET: Fisher’s exact test.

# Figure titles and legends

**Figure S1.** Study design

Legend: Study design depicting medication intake and time points of blood pressure, heart rate, cortisol, aldosterone, and DHEA-S measurements.

**Figure S2**. Systolic blood pressure

Legend: Mean systolic blood pressure (SE) for (A) both groups across conditions, (B) healthy controls for each condition, and (C) depressed patients for each condition.

**Figure S3.** Diastolic blood pressure

Legend: Mean diastolic blood pressure (SE) for (A) both groups across conditions, (B) healthy controls for each condition, and (C) depressed patients for each condition.

**Figure S4.** Heart rate

Legend: Mean heart rate (SE) for (A) both groups across conditions, (B) healthy controls for each condition, and (C) depressed patients for each condition.

**Figure S5.** Mood assessment

Legend: Mean mood measurements (SE) for both groups and for all three measurement time points. VAMS = visual analogue mood scale.
