# Supplementary material for: Steroid hormone secretion after stimulation of mineralocorticoid and NMDA receptors and cardiovascular risk in patients with depression
Source: Transl Psychiatry. 2020 Apr 20;10:109. doi: 10.1038/s41398-020-0789-7 (PMC7171120; doi:10.1038/s41398-020-0789-7)
Supplement: Supplementary file 2 — Table S1 [file 41398_2020_789_MOESM2_ESM.docx]

**Table S1**. Sample characteristics separated for each condition

|  | Placebo | FLU | DCS | FLU + DCS | Statistics |
| --- | --- | --- | --- | --- | --- |
| *n* | 58 | 58 | 58 | 58 |  |
| Age, mean (*SD*) | 35.6 (13.3) | 35.3 (12.5) | 34.3 (13.4) | 34.0 (13.9) | *F*(3,228) = 0.2, *p* = .90 |
| Women, *n* (%) | 40 (69%) | 40 (69%) | 48 (83%) | 54 (93%) | *χ²*(3) = 14.2, *p* < .01 |
| Education years | 12.0 (1.3) | 11.9 (1.3) | 12.0 (1.3) | 11.8 (1.3) | *F*(3,228) = 0.4, *p* = .80 |
| BMI | 24.0 (4.1) | 23.7 (4.1) | 23.9 (3.8) | 23.3 (3.6) | *F*(3,228) = 0.3, *p* = .80 |
| Smoker | 14 (24%) | 7 (12%) | 12 (21%) | 11 (19%) | *χ²*(3) = 2.9, *p* = .41 |
| Hormonal contraception | 8 (14%) | 7 (12%) | 10 (17%) | 13 (22%) | *χ²*(3) = 4.4, *p* = .93 |
| HAMD | 11.9 (10.9) | 10.9 (9.4) | 11.8 (10.7) | 11.5 (10.3) | *F*(3,228) = 0.1, *p* = .96 |
| BDI | 12.8 (12.7) | 13.3 (13.4) | 14.3 (14.4) | 13.7 (14.2) | *F*(3,228) = 0.1, *p* = .95 |

Legend: FLU = Fludrocortisone; DCS = D-cycloserine; BMI = Body mass index; HAMD = Hamilton Ratings Scale for Depression; BDI = Beck Depression Inventory; Values represent mean (SD) or n (%).
