# Supplementary material for: Steroid hormone secretion after stimulation of mineralocorticoid and NMDA receptors and cardiovascular risk in patients with depression
Source: Transl Psychiatry. 2020 Apr 20;10:109. doi: 10.1038/s41398-020-0789-7 (PMC7171120; doi:10.1038/s41398-020-0789-7)
Supplement: Supplementary file 3 — Table S2 [file 41398_2020_789_MOESM3_ESM.docx]

**Table S2**. Depression characteristics for the whole sample and four conditions

|  | In total | Placebo | FLU | DCS | FLU + DCS | Statistics |
| --- | --- | --- | --- | --- | --- | --- |
| *n* | 116 | 29 | 29 | 29 | 29 |  |
| Depression severity |  |  |  |  |  | FET (*p* = .29) |
| Mild | 16 (14%) | 4 (14%) | 5 (17%) | 2 (7%) | 5 (17%) | FET (*p* = .64) |
| Moderate | 88 (76%) | 21 (72%) | 23 (79%) | 21 (72%) | 23 (79%) | *χ²*(3) = 0.8, *p* = .86 |
| Severe | 12 (10%) | 4 (14%) | 1 (4%) | 6 (21%) | 1 (4%) | FET (*p* = .10) |
| Depression course |  |  |  |  |  | FET (*p* = .93) |
| First episode | 16 (14%) | 2 (7%) | 4 (14%) | 5 (17%) | 5 (18%) | FET (*p* = .64) |
| Recurrent | 49 (42%) | 14 (48%) | 12 (41%) | 11 (38%) | 12 (41%) | FET (*p* = .91) |
| Persistent | 51 (44%) | 13 (45%) | 13 (45%) | 13 (45%) | 12 (41%) | *χ²*(3) = 0.1, *p* = .99 |
| Depression specifier |  |  |  |  |  | FET (*p* = .95) |
| Melancholic | 31 (14%) | 10 (35%) | 6 (22%) | 8 (27%) | 7 (24%) | *χ²*(3) = 1.3, *p* = .74 |
| Anxious | 67 (59%) | 16 (55%) | 16 (59%) | 17 (59%) | 18 (62%) | *χ²*(3) = 0.3, *p* = .96 |
| Atypical | 16 (27%) | 3 (10%) | 5 (19%) | 4 (14%) | 4 (14%) | FET (*p* = .85) |

Legend: FLU = Fludrocortisone; DCS = D-cycloserine; in the fludrocortisone condition, specifier information was missing for two participants. FET: Fisher’s exact test.
