# Supplementary figures and images for: Steroid hormone secretion after stimulation of mineralocorticoid and NMDA receptors and cardiovascular risk in patients with depression
Source: Transl Psychiatry. 2020 Apr 20;10:109. doi: 10.1038/s41398-020-0789-7 (PMC7171120; doi:10.1038/s41398-020-0789-7)

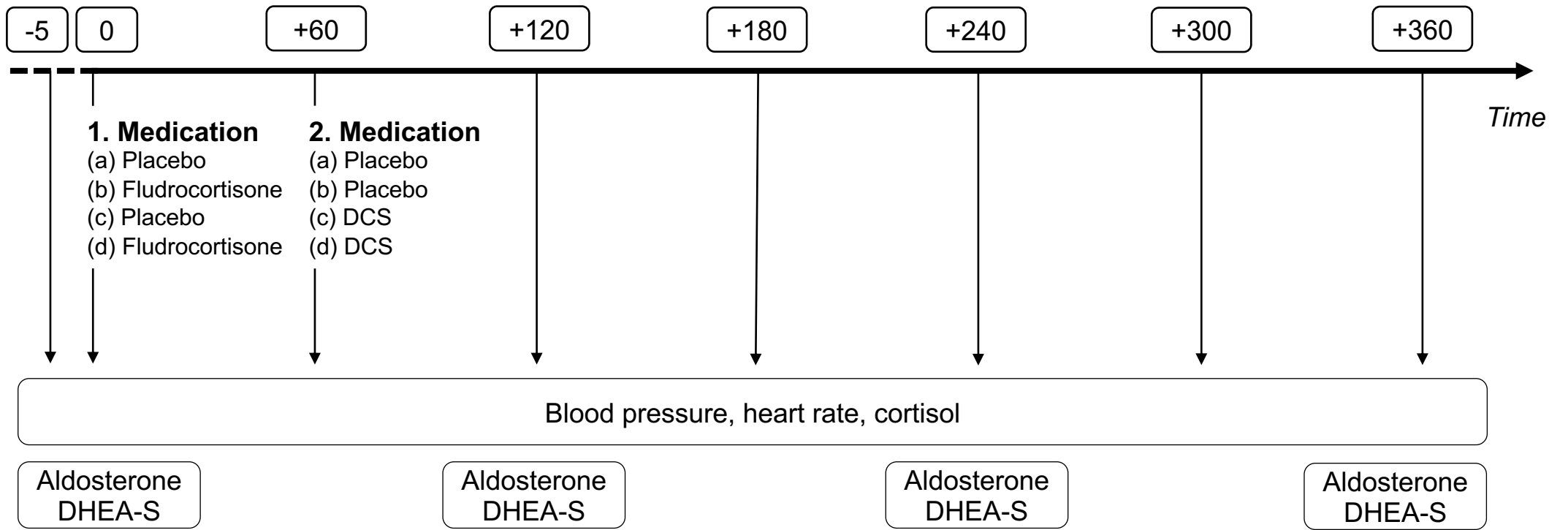

Supplement: Supplementary file 4 — Figure S1 [file 41398_2020_789_MOESM4_ESM.pdf]
